# Protocol for a 24-Week Randomized Controlled Study of Once-Daily Oral Dose of Flax Lignan to Healthy Older Adults

**DOI:** 10.2196/resprot.6817

**Published:** 2017-02-03

**Authors:** Jane Alcorn, Susan Whiting, Navita Viveky, Yunyun Di, Kerry Mansell, Sharyle Fowler, Lilian Thorpe, Ahmed Almousa, Pui Chi Cheng, Jennifer Jones, Jennifer Billinsky, Thomas Hadjistavropoulos

**Affiliations:** ^1^ College of Pharmacy and Nutrition University of Saskatchewan Saskatoon, SK Canada; ^2^ Division of Gastroenterology College of Medicine University of Saskatchewan Saskatoon, SK Canada; ^3^ Community Health and Epidemiology College of Medicine University of Saskatchewan Saskatoon, SK Canada; ^4^ Department of Psychology Faculty of Arts University of Regina Regina, SK Canada

**Keywords:** flax, lignan, inflammation, oxidative stress, clinical trial, older adults

## Abstract

**Background:**

Increased oxidative stress and inflammation are associated with aging, and contribute to an increased risk of chronic disease in older adults. Flaxseed lignans demonstrate antioxidant and anti-inflammatory activity, but their ability to reduce oxidative stress and inflammation markers in older adult populations has received limited investigation.

**Objective:**

This is a chronic intervention trial of community-dwelling healthy older adults to examine the effects of a flaxseed lignan (secoisolariciresinol diglucoside; SDG) enriched supplement (BeneFlax) compared to a placebo. The primary aim was to demonstrate the safety of BeneFlax and confirm its anti-inflammatory efficacy on markers of oxidative stress and inflammation, and subsequent functional outcomes, including those associated with its anti-inflammatory efficacy. A secondary aim was to determine flaxseed lignan metabolite concentrations in blood.

**Methods:**

A double-blind randomized clinical trial was conducted. Subjects were healthy community-dwelling adults aged 60-80 years. Testing was performed at baseline, 8, 16, and 24 weeks. The 24-week intervention consisted of 600 milligrams (mg) of SDG daily or an equivalent amount (volume) of placebo. All participants received 1000 international units of vitamin D to ensure adequate vitamin D status. Measurements consisted of blood pressure, hematology, and tolerability for safety assessments; blood oxidative stress and inflammatory biomarkers for efficacy; and cognition, muscle strength, and pain as functional outcomes. Secondary endpoints of plasma levels of lignan metabolites were analyzed by mass spectrometry. Other tests, such as bone turnover markers and fecal levels of flax cyclolinopeptides, will be performed at a later date.

**Results:**

Thirty-two participants were recruited (19 intervention and 13 control) and all completed the trial. Numerous Health Canada-imposed exclusion criteria limited recruitment success. Analyses are ongoing, but the baseline data available for a number of parameters indicate no differences between treatment groups. Safety measures (vital signs) did not change from baseline and were not significantly different between treatment and placebo groups at 24 weeks.

**Conclusions:**

Preliminary results indicate that no safety concerns are associated with administering 600 mg SDG for 24 weeks to adults between the ages of 60 and 80 years.

**Trial Registration:**

Clinicaltrials.gov NCT01846117; https://clinicaltrials.gov/ct2/show/NCT01846117 (Archived by WebCite at http://www.webcitation.org/6nlDZNjmA)

## Introduction

Oxidative stress and inflammation are associated with a number of chronic diseases that are common among older adults [1,2]. Decreasing these processes may ameliorate problems associated with aging, such as hypertension and inflammation, which promote the development of vascular dementia and Alzheimer’s disease (resulting in cognitive impairment) [3], and muscle wasting promoted by proinflammatory cytokines such as interleukin-6 (IL-6) and tumor necrosis factor-alpha (TNF-α) [4]. Increased oxidative stress also appears to be an early instigator of metabolic syndrome [5]. Evidence from animal and human studies demonstrates that flax lignans, such as secoisolariciresinol diglucoside (SDG), may delay the development of diseases associated with inflammation (ie, type 2 diabetes [6]), decrease hypertension [7,8], and lower serum cholesterol levels [9], among other actions. SDG supplementation in adults is associated with decreased levels of cholesterol and glucose in hypercholesterolemic individuals [9], reduced concentrations of C-reactive protein [10], and decreased metabolic syndrome composite score [7].

While older individuals would be expected to benefit more from anti-inflammatory compounds, most studies have focused on adults <60 years of age [9]. Consequently, limited information is available in seniors, and studies are necessary to confirm the safety and efficacy of anti-inflammatory compounds in this subpopulation of older adults. When SDG was tested in younger adults in a human clinical trial, there were no safety concerns associated with intake of 600 milligrams (mg) SDG, ingested for 8 weeks in participants between 53 and 58 years of age [9]. We also previously conducted two trials of SDG. One trial used a dose of 500 mg SDG in older (60-70 years of age) community-dwelling adults [7] but that trial had exercise in both the treatment and control groups, so assessment of SDG-only treatment was not possible. We then conducted a trial in older residential care (nursing home) adults aged 60-80 years, but that trial used a dose of only 300 mg SDG, as per Health Canada permission, and suffered from low recruitment (due to multiple exclusion criteria) and low retention (due to subject frailty) [11].

The present study was designed to examine whether consumption of a pharmacological dose (ie, 600 mg/day) of the flax lignan SDG for approximately 6 months (which was predicted to reduce oxidative stress and inflammation [12]), would show evidence of efficacy and safety in community-dwelling healthy older adults. A battery of biochemical and functional tests was applied. A 1000 international unit (IU) vitamin D supplement was given to all participants to ensure similar vitamin D status, in order to avoid confounding effects of differing status. The hypothesis being tested was that consumption of SDG, in persons with adequate vitamin D status, will decrease oxidative stress and associated inflammation, and improve secondary measures of function at the 6-month time point. In addition, data regarding blood lignan metabolites was gathered.

## Methods

### Intervention and Participant Recruitment

A 24-week double-blind randomized clinical trial was conducted, in which the intervention consisted of 600 mg flax lignan SDG daily or an equivalent amount of placebo. Participants were healthy community-dwelling men and women between the ages of 60 and 80 years, living in Saskatoon, Canada. The study was conducted in 2013-2014. Exclusion criteria included: age below 60 or above 80 years at initiation of the study; living in long term care (nursing) homes; individuals at risk of hypotension or with symptomatic hypotension; fasting hypoglycemia; unstable diabetes, or diabetics taking insulin; current cancer or diagnosed with cancer in the past 2 years; women with an immediate family history or personal history of breast cancer or ovarian cancer; significant liver (or other gastrointestinal) disorder, including inflammatory bowel disease; significant kidney disorder; unstable or severe cardiac disease, recent myocardial infarction, or stroke (either in past 6 months or significantly affecting physical mobility); unstable other medical disease including, but not limited to, pulmonary disorder, epilepsy, and genitourinary disorder; migraine with aura within the last year (as this is a risk factor for stroke); current diagnosis of a bleeding condition, or at risk of bleeding; significantly immunocompromised; current use of hormone replacement therapy (except thyroid medication); current use of warfarin, clopidogrel, ticlopidine, dipyridamole, or their analogues; intolerances or allergies to flax or vitamin D; allergy to whey (placebo); surgery within the last six months; and participation in any other clinical trial with an investigational agent within one month prior to randomization.

**Figure 1:**
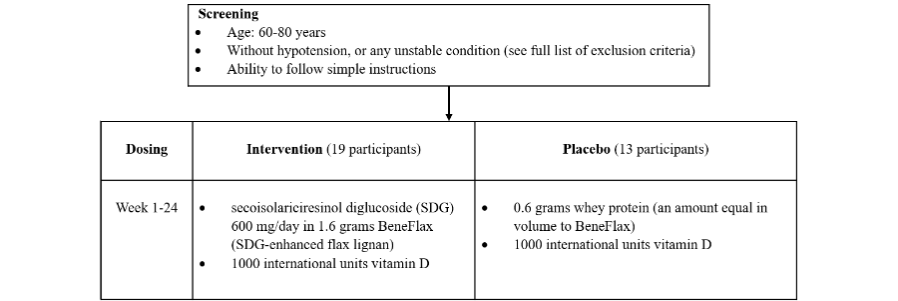
Schematic of trial design, procedures, and stages.

**Figure 2:**
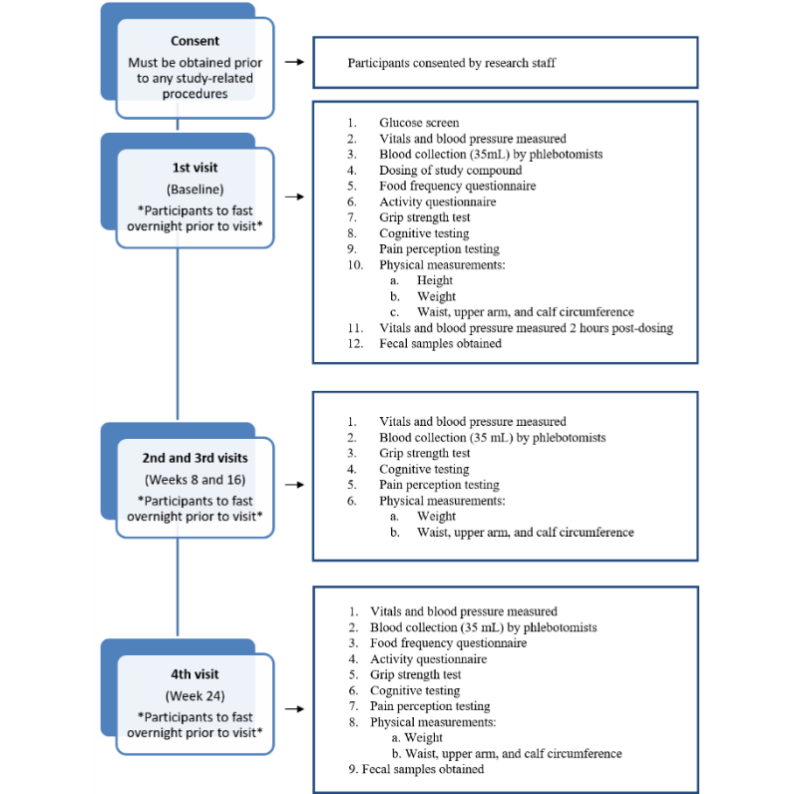
Flow chart of events for study.

Recruitment of participants was undertaken using posters and newspaper advertisements. Study posters were displayed in local hospitals, on the University of Saskatchewan campus, and in several senior residences. The contact information of the study coordinator was provided. Interested volunteers called the study coordinator, who reviewed inclusion and exclusion criteria over the phone. Volunteers who met the inclusion criteria were asked to visit the study coordinator’s office at the College of Pharmacy and Nutrition, University of Saskatchewan, Saskatoon (one time only) where inclusion and exclusion criteria were again reviewed and information in the consent form (10-page document) was explained, including purpose (Figure 1) and procedures (Figure 2), along with the possible risks and benefits of the study. Volunteers were given sufficient time to think about their participation. All volunteers were given the opportunity to ask questions and were made aware that they could withdraw from this study at any time for any reason. At the end of the consent process, volunteers gave permission to use and disclose their deidentified information that was collected for research purposes. A signed copy of the consent form was provided to each study participant. After participants signed up for the study, a study number was assigned and participants were given a map of the Saskatchewan Centre for Patient-Oriented Research (SCPOR) facility. Participants were also given a toilet hat and instructions regarding the collection of fecal samples for baseline. All study visits took place at the SCPOR facility located at City Hospital in Saskatoon, Canada.

### Study Procedures

The details of tests and timing are described in Figure 2. After obtaining informed consent from the participants, research staff collected demographic information, including age, date of birth, race, sex, and personal health number (required for blood collection). Screening for inclusion and exclusion criteria was done before each visit (ie, baseline, week 8, week 16, and week 24). The list of medications (including vitamins, supplements, natural health products, over the counter medications, and prescription medications; total daily dose if regular or individual dose if used as needed) was obtained from all study participants at baseline, and changes to medications were enquired at every consecutive visit with the review of inclusion/exclusion criteria.

**Figure 3:**
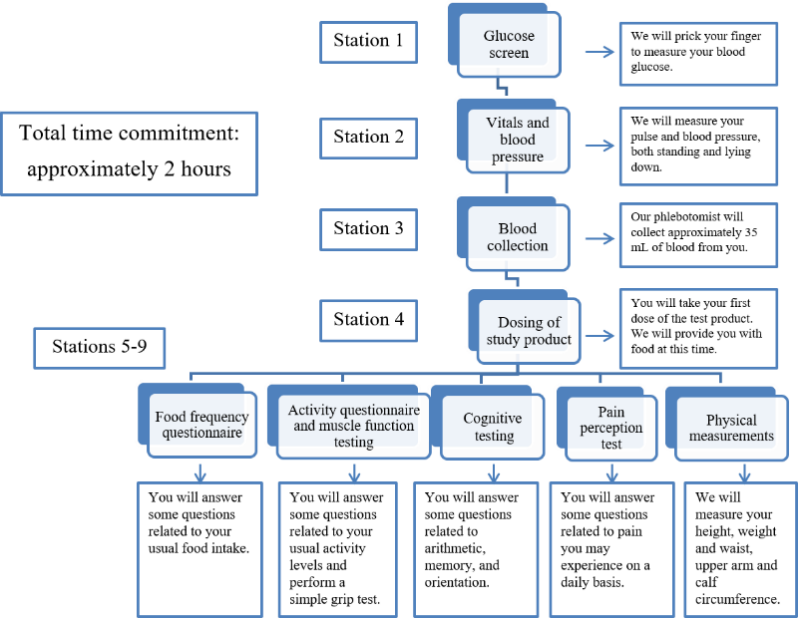
The flowchart of procedures that was handed to participants at each visit. Visit 1 had an additional reminder regarding the 2-hour post dosing measures.

A flowchart of the study procedures corresponding to visits 1 through 4 is shown in Figure 2. Clinical facilities and lounge areas at SCPOR were available for the study. Some measures were assessed at all visits while others were completed only at visit 1 (eg, the two-hour postdosing measurements), and fecal samples were collected only at visits 1 and 4. Figure 3 shows instructions given to the participant upon his or her arrival at the study center. Nine stations were set up in four different areas (to allow for privacy) and participants went from station 1 to station 4 in order, and then stations 5 through 9 in any order. At the end of each visit, a checklist was signed off by research staff to ensure that all study tests and procedures were completed. Upon completion of the checklist, participants were given a light lunch.

### Randomization and Blinding

We aimed to recruit a total of 60 participants (30 per treatment group) per protocol analysis. We calculated that a blood pressure change of 10 mmHg (inducing hypotension as a safety concern) would require 30 participants per group. However, recruitment proceeded slowly; thus, we started the study with 32 participants, with 19 in the intervention group and 13 in the control group. This distribution was generated randomly to allow for an equitable sex distribution.

BeneFlax or placebo were administered in a double-blind fashion. Only the pharmacist, who used a computer-aided randomization system, knew group assignments; all personnel performing the data collection and analyses were blind to group assignment. The pharmacist kept a secure copy of the randomization codes during the study. The code was broken only after results were analyzed. Some tests have yet to be been run (ie, some of the cytokine markers of inflammation, bone turnover markers, and fecal cyclolinopeptides).

### Accountability Procedures

The placebo (whey powder, Natural Factors) was purchased from a health food store in Saskatoon, Canada. The flax lignan supplement BeneFlax was shipped to the College of Pharmacy and Nutrition Saskatoon, Canada (a secured facility) from Archer Daniels Midland (Natural Health Products File # OF2-31-3-13412-2-4), and stored at –20°C. The analyzed content of SDG in BeneFlax was measured prior to the study (samples were shipped to Archer Daniels Midland for quantitative analysis). The packets of BeneFlax and whey were prepared by the research staff following safe food preparation procedures. Study compounds were sent to a designated pharmacy where the pharmacist oversaw the dispensing of products into packets, which were labelled and packaged into 12-week supplies in child-proof amber containers, with instructions to store the containers in the fridge. The pharmacist kept accurate records of the study compounds dispensed, with identification of the participant to whom they were dispensed and the date of the dispensing. During the course of the study all unused study compounds were returned to the researchers. The 1000 IU vitamin D supplements were purchased from the pharmacist and were dispensed in their original packaging.

### Intervention

This study used a two-group randomized design with an intervention group and a control group. The intervention group received 1.6 grams of SDG-enriched food-grade flaxseed lignan complex BeneFlax containing 600 mg SDG, plus 1000 IU vitamin D, while the placebo control group received 0.6 grams of whey protein (similar in volume to the intervention compound) plus 1000 IU vitamin D. Vitamin D tablets were provided separately. The daily dose could not be delivered in one packet, so instructions indicated that two packets were to be consumed per day. Each packet contained 0.8 grams of BeneFlax or 0.3 grams of placebo (a volume equivalent to the volume of BeneFlax given). The product was taken at the same time each day (eg, always with breakfast or always with dinner). Either compound (lignan or whey powder) could be added to a tablespoon of applesauce or equivalent food.

Usual medications were allowed except for warfarin, clopidogrel, ticlopidine, dipyridamole (or their analogues), and female hormone replacement therapy. Participants took at minimum 1000 IU vitamin D and were allowed other multivitamin/mineral formulations containing vitamin D as part of their supplemental routine. Participants were also asked to refrain from consuming flax-containing foods throughout the 24-week period of the study. All participants were provided with a diary at baseline to assess compliance, medication use, and bowel health monitoring. The initial pages included a checklist, and participants were instructed to place a checkmark in a square to indicate if they took the study product and vitamin D for each day in the 24 weeks of the study period. The medication list included all prescriptions, natural health products, vitamins, and supplements that they were using at baseline, along with any that were added during the duration of this study. Bowel health monitoring was assessed by study participants making note of any changes that differed from usual bowel health. These changes included alterations in bowel movement frequency, consistency, or any discomfort felt in the gastrointestinal tract. The last section of the diary was used to keep record of any illnesses along with the date, duration of symptoms (eg, once; number of days), and symptoms (date symptoms started and date symptoms ended).

### Samples and Testing

Primary outcome measures were those of safety, related to 6-month administration of 600 mg SDG per day in healthy older adults. We included the reporting of clinical adverse signs and symptoms, vital signs, serum clinical chemistry, and hematology parameters for safety. SDG is known to be anti-inflammatory [10], so we also sought to confirm this property in older adults by examining the effects of flaxseed lignan supplementation on biomarkers of inflammation, risk factors of cardiovascular disease, and functional (ie, quality of life) indicators. Secondary outcomes included measuring plasma concentrations of lignans, fecal levels of flax cyclolinopeptides, and plasma levels of cyclolinopeptides.

Blood was collected on four visits: at baseline before taking the first dose of test product; and at weeks 8, 16, and 24. Phlebotomy staff at Saskatoon City Hospital collected a total of 35 milliliters (mL) of blood at each visit. Blood was collected in three 4.5 mL plasma separation tubes, two 10 mL tubes containing the anti-coagulant dipotassium ethylenediaminetetraacetic acid (K_2_ EDTA), and two 4 mL K_2_ EDTA tubes (or three 4 mL K_2_ EDTA tubes in the case of diabetic participants), and all tubes were placed on ice packs. After centrifugation for 10 minutes at 1500 revolutions per minute (rpm), plasma was aliquoted into clearly labelled microcentrifuge vials to a volume of 500 microliters (µL) and placed at –20°C immediately, and research staff recorded the number of vials aliquoted each time. Subsequently, samples were stored at –80°C until analysis.

Two fecal samples were collected: once at the beginning of the trial, and again at the 24-week visit at the end of the trial. All required supplies and instructions to collect fecal samples were provided to participants. Participants were informed that fecal collection was to be made on the first bowel movement of the day of visit (baseline and 24-week) or samples collected at an earlier date (if they could not produce a fecal sample on the morning) were placed in freezer at home and brought to SCPOR by participants. Fecal samples were labeled by research staff and stored at –20°C immediately. Subsequently, fecal samples were also stored at –80°C until analysis.

Blood analysis was primarily carried out by the Saskatoon Health Region through the company Gamma Dynacare. Gamma Dynacare analyzed the following at all four time points (unless stated otherwise): urea, creatinine, glucose, liver enzymes (alanine transaminase [ALT], aspartate transaminase [AST], alkaline phosphatase [ALP]), total bilirubin, total protein, albumin, total cholesterol, triglycerides, high density lipoprotein (HDL) cholesterol, low density lipoprotein cholesterol, total cholesterol to HDL ratio, total calcium, electrolytes (sodium, chloride, potassium), magnesium, prealbumin, complete blood count (platelets, hematocrit, hemoglobin, mean corpuscular hemoglobin, mean corpuscular volume, white blood cell count), plasma C-reactive protein, and 25-hydroxyvitamin D. Research staff measured markers of oxidative stress, inflammation, and flaxseed lignan metabolites. Fecal samples will be analyzed for cyclolinopeptides by research staff.

A battery of functional tests was carried out. Cognitive function was assessed by research staff using the Mini-Mental State Examination, a clinical practice and research tool that takes 5-10 minutes to systematically assess mental status. The tool measures five areas of cognitive functionality: orientation, registration, attention and calculation, recall, and language, with 11 predefined questions. The maximum score is 30 and a score of 23 or lower is indicative of cognitive impairment [13]. Pain was assessed by research staff using the Brief Pain Inventory (BPI) Short Form [14]. Using BPI, participants rated the severity of their pain along with the degree to which it interfered with feeling and function.

Physical function was assessed by research staff via grip strength, using a Baseline Hydraulic Hand Dynamometer (Fabrication Enterprises Incorporated, White Plains, NY). Participants were seated comfortably with their upper arm in a normal neutral position alongside the body, and the elbow joint bent at 90°. The Hand Dynamometer was set to position 3 (or position 2 for females). The handgrip device was positioned vertically in the hand during the contractions (no rotating or twisting). Participants were instructed to squeeze the handle as hard as possible for a count of 3 (3-second long *isometric* contraction). Each participant’s dominant side was tested first and 3 maximal repetitions with 30 seconds of rest between attempts were collected. Grip strength testing was completed on one hand before switching to the nondominant hand. Participants were not informed of the scores on each repetition until all repetitions were completed. If the participant was not able to complete the test, it was recorded as *unable*. If the participant could not complete the test due to a medical reason (ie, having had a past stroke), details were recorded in comments. Measurements were recorded to the nearest kilogram (kg). Height, weight, and circumference of the waist, mid-upper right arm, and right calf were measured by research staff using standard procedures [15].

The University of Saskatchewan version of the Block Food Frequency Questionnaire, modified to reflect Canadian fortification and to collect data on flax use, was administered to participants at each visit [7]. Activity was assessed by brief four-item queries of usual leisure-time exercise habits using the Godin Leisure-Time Exercise Questionnaire. Participants were asked during a typical 7-day period (one week), how many times (ie, each time = 1 unit) on average they did strenuous (eg, one’s heart beating rapidly) **,** moderate (not exhausting), and mild (minimal effort) exercise for more than 15 minutes. Scoring in Godin Leisure-Time Exercise Questionnaire was based on a cut point at 24 units, which represented the cumulative score of two intensities: strenuous and moderate. A score of >24 units is categorized as active with substantial benefits, 14-23 units are moderately active with some benefits, and <14 units indicate insufficiently active with less substantial/low benefits [16].

### Other Efficacy Measures

C-reactive protein was measured by Pathology and Laboratory Medicine, Saskatoon Health Region. Other oxidative stress measurements (plasma malondialdehyde) and proinflammatory measurements (IL-6, TNF-α) were measured by the research staff using kits purchased from Cayman (Ann Arbor, Michigan, US). All other solvents were specified as mass spectrometry grade and all other chemicals were reagent grade. Plasma 25-hydroxyvitamin D was measured in-hospital for vitamin D assessment.

To further understand the pharmacology of SDG, plasma levels of SDG metabolites (secoisolariciresinol, enterodiol, enterlactone) were analyzed using various methods. Plasma trough concentrations of flaxseed lignan metabolites were measured to provide important information regarding lignan levels with chronic oral administration of a pharmacological dose of SDG contained in BeneFlax (approximately 38% SDG). Participant plasma samples were collected and stored as stated above. Stock solutions (1 mg/mL) of lignan metabolites and their respective stable isotope-labelled internal standards (Toronto Research Chemicals) were prepared in methanol and stored at –20°C. Working solutions were prepared by serial dilution of the stock solution to produce a standard calibration curve of 0.2-50 nanograms (ng)/mL for enterolactone and enterodiol, and 1-50 ng/mL for secoisolariciresinol. Previous studies have indicated that SDG is not absorbed and therefore was not analyzed. Quality control (QC) standards were prepared for acceptance criteria of the analytical assay. Calibration and QC samples were prepared on ice for each day of sample analysis. A linear least-squares regression analysis using 1/X^2^as weighting factor was conducted to determine slope, intercept, and coefficient of determination (r^2^) to demonstrate linearity of the method.

The sample extraction procedure involved the addition of 30 µL of internal standard and 4 mL diethyl ether to 300 µL of thawed plasma samples, and the mixture was shaken vigorously for 10 minutes. Samples were centrifuged at 2500 rpm for 5 minutes to separate the organic layers and transferred to –80°C to freeze the aqueous layer. The organic phase was then transferred to a glass tube and dried by rotary vacuum. Samples were reconstituted in 150 µL of mobile phase (85:15 solvent ratio [A:B] containing 0.1% formic acid) and filtered through Whatman Mini-UniPrep Syringeless Filter vials. For measuring the total lignans in plasma (free and conjugated lignans), 300 µL plasma and 60 µL beta-glucuronidase were added to 300 µL sodium acetate buffer (0.1 molar, pH 5.0) and incubated at 37°C for 4 hours before proceeding to the extraction procedure.

For high-performance liquid chromatography, 5 μL of sample was injected onto a Porshell 120 EC-C18 2.1 x 50 millimeter (mm), 2.7 micrometer (μm) column and 2.1 x 5 mm, 2.7 μm guard column (Agilent Technologies) with the column temperature set at 20°C. Samples were separated using an Agilent series 1200 binary pump (Agilent Technologies, Mississauga, Ontario, Canada) with an online degasser and auto sampler set at 4°C. Analytes were detected with an AB Sciex API 4000 Q-TRAP mass spectrometer (AB Sciex, Concord, Ontario, Canada). The mobile phase was 0.1% formic acid in liquid chromatography-mass spectrometry (LC-MS) grade water (Solvent A) and LC-MS grade acetonitrile (Solvent B). The flow rate was set to 250 µL/min. Samples were separated using the 10-minute gradient method. The mobile phase was started with 85:15 A:B at 0 minutes and dropped to 50:50 A:B from 0 to 1.5 minutes. The gradient continuously changed to 5:95 A:B from 1.5 minutes to 2.5 minutes and remained the same to 4.5 minutes. The mobile phase was quickly returned to 85:15 A:B from 4.5 minutes to 5 minutes and held for another 5 minutes to equilibrate the column.

LC-MS was performed in the negative ion mode. The AB Sciex 4000 Q-TRAP mass spectrometer utilized a curtain gas pressure of 10 pounds per square inch and GS1 and GS2 parameters were set at 50 pounds per square inch. The ionspray voltage was set at 4500 V and the temperature of the electrospray ionization source interface was maintained at 700°C. The mass spectrometer utilized multiple reaction monitoring to quantify the analytes, by using the transition of mass (M) and charge number (z) such that [M]+ (m/z 361.019>164.800; declustering potential of 90, collision energy of 36, collision cell exit potential of 11) for secoisolariciresinol, transition of [M]+ (m/z 301.000>253.000; declustering potential of 95, collision energy of 32, collision cell exit potential of 5) for enterodiol, and transition of [M]+ (m/z 297.000>189.000; declustering potential of 90, collision energy of 30, collision cell exit potential of 7) for enterolactone. The peak areas were summed through use of Analyst Software. The ratio of peak areas of lignan metabolites to their respective internal standards were plotted against the nominal concentrations to construct the calibration curve. Analytical method validation was performed in accordance with the United States Food and Drug Administration (FDA) guidelines [17]. The assay was specific and linear, extraction efficiency ranged from 50% to 72%, and intraday and interday precision and accuracy of the method was within 15%.

### Safety Parameters

Urea, creatinine, total bilirubin, platelets, hematocrit, hemoglobin, mean corpuscular hemoglobin, mean corpuscular volume, white blood cell count, total calcium, glucose, liver enzymes (AST, ALT, ALP), total protein, albumin, lipids, glycated hemoglobin for diabetic participants, and electrolytes were measured for safety assessment. Vital signs, including blood pressure, heart rate, and respiration rate were also assessed, with a copy of these results sent to the participant’s physician. Blood pressure using a standard hospital sphygmomanometer was measured as resting blood pressure for all participants, in addition to standing blood pressure using the blood pressure monitor. All vital signs (except respiratory rate) were taken when study participants were either lying down or standing. Study participants would lie quietly for 3-5 minutes prior to blood pressure and pulse readings being taken; they then stood for 1 minute prior to blood pressure and pulse being taken again. If blood pressure measurements exceeded the inclusion criteria of *mild* hypertension (140-159 mmHg/90-100 mmHg) or fell below cutoffs for systolic hypotension (systolic blood pressure <80 mmHg) or orthostatic hypotension (reduction of systolic blood pressure of at least 20 mmHg or diastolic blood pressure of at least 10 mmHg within 3 minutes of standing after restful sitting for at least five minutes) at visit 1, participants would have been excluded; however, no participants met these extreme cut-offs. During the study, hypoglycemia, systolic hypotension, and orthostatic hypotension episodes were used as indicators of adverse effects. Respiration rate was measured by the number of breaths in one minute by counting how many times the chest rose.

A research assistant reviewed the results and flagged any values that were outside of the normal range; values were signed off by a designated physician, and those of concern were discussed with the principal investigator or study physician. Diabetic participants had fasting glucose monitored for hypoglycemia using Rapid Response test strips on Rapid Response blood glucose meter (BTNX Inc, Markham, Ontario, Canada).

### Ethics

Ethical approval was obtained from the University of Saskatchewan/University of Regina Ethics Review Board for Biomedical Research in Human Subjects. Approval from Health Canada was obtained for use of BeneFlax, a natural health product approved by both the FDA and Health Canada. This study is one of a series of studies that were supported by a Team Grant from the Saskatchewan Health Research Foundation to the University of Regina. This particular substudy was undertaken at the University of Saskatchewan and fell under the insurance of that institution.

### Adverse Events Monitoring

Adverse events were recorded throughout the study. An *adverse event* was defined as any untoward medical occurrence in a patient or clinical investigation participant that was administered a pharmaceutical product. All research team members in contact with participants were responsible for noting adverse events, which were reported by the participants. Participants were advised to communicate the adverse event at the time of occurrence to the Physician Responsible for Trial Site Medical Decisions during office hours, or to the on-call study physician if outside of office hours. The 24-hour contact numbers for study physicians were made available to all participants. Adverse events were documented with clinical details, as well as the date, start and stop time of the event, and severity. All actions taken and outcomes were documented. All potentially adverse experiences, including illnesses, unpleasant symptoms, and falls and injuries were charted. Upon the occurrence of a serious adverse event, Internal Serious Adverse Event Reporting Form from University of Saskatchewan (page 39) and Adverse Reaction Report Form for Clinical Trials from Health Canada (page 41) forms were to be filled; however, none occurred during this clinical trial.

### Statistics

The data will be descriptively analyzed using frequencies, means, and standard deviations. The data will be assessed for potential outliers or extreme observations, and for adherence to the assumptions underlying the analytic model. The data will be analyzed using a random-effects regression model for repeated measures data. A random-effects model is an appropriate and valid choice for these data given the potential for a high proportion of missing observations due to loss to follow-up, as a random-effects model does not require complete data and does not rest on the stringent assumptions regarding the covariance structure of the data that underlies the repeated measures analysis of variance (ANOVA). Any deviations from the original statistical plan will be described and justified in the final report. Model effects will include group (intervention, control) and time (baseline, 8, 16, and 24 weeks). Analyses will be conducted using body weight, blood pressure, age, and body mass index (kg/m^2^) as potential confounding covariates in the model. Likelihood ratio tests will be used to test for differences between treatment and control groups at each measurement occasion and/or between baseline and 24 weeks. A Cronbach alpha level of 0.05 will be used as the level of significance.

Data will be analyzed on an intent-to-treat basis; an attempt will be made to remeasure participants that did not adhere to supplementation. Analyses will also be performed by received dose (ie, analysis by the actual amount of supplement consumed by subtracting missed doses) using the diary information. A consort checklist for this study was completed according to established protocol and is presented as Multimedia Appendix 1 [18].

## Results

This trial was started on May 8, 2013. We screened 173 potential participants (92 females, 81 males) who responded to advertisements that listed inclusion criteria of 60-80 years of age, and “healthy.” There were 34 potential participants (16 females, 18 males) who consented to be in the study; however, after consenting two participants (one male and one female) withdrew. The 32 participants remaining in the study came to all four visits except one participant who missed the 16-week time point. Of the data from 128 total possible visits from 32 participants, we obtained data from 127 visits. Information on study participants is provided in Table 1, showing no differences between those randomized to intervention with those randomized to control treatment, using one-way ANOVA.

Here we report on some of the safety parameters. Systolic blood pressure, diastolic blood pressure, and heart rate measurements were completed in duplicate for all participants at each visit. Table 2 shows blood pressure (systolic and diastolic), heart rate, and respiration rate two hours after the BeneFlax or placebo was administered for the first time, and after 24 weeks of chronic ingestion (in fasting subjects). There was no significant change in any of these parameters with treatment, using one-way ANOVA.

**Table 1:** Study participants at baseline.

Parameter	BeneFlax	Placebo
Total (males, females)	19 (10, 9)	13 (7, 6)
Age in years, mean (SD)	67.9 (5.2)	68.1 (4.7)
Body mass index, mean (SD)	26.0 (3.3)	28.8 (5.0)
Systolic blood pressure, mean (SD)	129.5 (23.6)	138.3 (19.9)

**Table 2:** Vital signs after acute (2-hour) and chronic (24-week) ingestion of BeneFlax or placebo.

Outcome	Treatment	n	Baseline	2-hour	24-week	Range
Systolic blood pressure, mean (SD)	BeneFlax	19	130 (24)	136 (20)	132 (14)	100-178
	Placebo	13	138 (10)	134 (21)	138 (21)	99-167
Diastolic blood pressure, mean (SD)	BeneFlax	19	79 (10)	80 (9)	79 (9)	64-109
	Placebo	13	77 (6)	74 (5)	76 (7)	64-89
Respiratory rate, mean (SD)	BeneFlax	19	16 (5)	13 (3)	13 (3)	7.0-24
	Placebo	13	15 (4)	15 (4)	14 (3)	9.0-24
Heart rate, mean (SD)	BeneFlax	19	63 (9)	66 (7)	69 (11)	47-88
	Placebo	13	64 (9)	67 (7)	63 (11)	49-86

## Discussion

Data analyses for this intervention trial are ongoing and flaxseed lignan metabolite measurements are underway. Results and findings will be reported in several publications. In terms of further analyses, the determination of the differences in the inflammatory and oxidative stress markers between the SDG- and placebo-supplemented group are in progress. This research will contribute to the literature on the efficacy and safety of SDG supplementation in healthy older adults. Data from other tests such as grip strength, pain measures, activity, anthropometrics, and cognitive testing will help in better understanding the effects of flaxseed lignan supplementation on functionality. Associations with oxidative stress and inflammation will be made.

In terms of safety, our data (Table 2) are compatible with our other studies in which we reported that SDG supplementation (300 mg/day of BeneFlax) in a very frail, complex patient population 60-80 years of age caused no significant adverse outcomes [11], and that 543 mg daily for 6 months produced no incidents of hypoglycemia or hypotension among participants 49-87 years of age [19].

Compliance with the Flax Product Study compound was monitored with the participant diary. Although participants did not consistently return all used and unused product packets of Beneflax and vitamin D supplements, most returned diaries outlining the compliance to study test products, vitamin D, medication change, illnesses, and bowel health monitoring.

Strengths of the study included the commitment of participants who presented themselves at 127 of 128 visits (32 participants and 4 visits), which represents 99.2% compliance to the study. Conducting this trial at SCPOR was another strength, as this facility had adequate space and rooms for all tests and procedures, which were simultaneously performed by the research staff during the visits. The centrifuge for obtaining plasma was in place at SCPOR and Pathology and Laboratory Medicine, Saskatoon Health Region was situated in the hospital as well, for immediate transfer of blood samples. The equipment used at SCPOR, such as blood pressure monitors and weighing scales, were calibrated by the technicians there, providing accuracy and precision to the tests. Also, SCPOR is located in the downtown area, making it easily accessible to the study participants. All tests and procedures performed by research staff were conducted using standard operating procedures; training was provided to staff.

The foremost study limitation and challenge was the recruitment of healthy older adults due to our extensive exclusion criteria. Of the 173 potential participants initially screened by research staff, only 34 participants met the inclusion/exclusion criteria, representing approximately 20% of the potential participants. After the withdrawal of two participants, we had only 32 final participants. Our previous work [11,19] demonstrated the safety of BeneFlax in older healthy and frail populations, therefore the inclusion and exclusion criteria could be revised/reviewed in further clinical trials of BeneFlax in older healthy and frail populations for better recruitment, and to allow for larger scale clinical trials.

### Conclusion

We are comparing flax lignan to a placebo (whey powder) to examine whether a dietary intervention (ie, flaxseed lignan-enriched product) might decrease oxidative stress and inflammation in older adults. This intervention consisted of 600 mg of the flaxseed lignan SDG, taken daily for 24 weeks in healthy older adults. SDG is broken down in the gastrointestinal tract to produce the health benefits of flax. Results from this study will demonstrate whether SDG supplementation decreases oxidative stress and inflammation in community-dwelling healthy older adults. These findings might help in maintaining/improving functionality markers such as cognition, muscle strength, and other inflammation-associated problems of aging.

To the best of our knowledge this is the first study testing the efficacy and safety of flaxseed lignan in community-dwelling healthy older adults. Our findings will contribute significantly to the knowledge base on flaxseed lignan safety and efficacy.

## Appendix

Multimedia Appendix 1 CONSORT-EHEALTH checklist V1.6.1 [18].

## Abbreviations

ALP: alkaline phosphatase
ALT: alanine transaminase
ANOVA: analysis of variance
AST: aspartate transaminase
BPI: Brief Pain Inventory
FDA: Food and Drug Administration
HDL: high density lipoprotein
IL-6: Interleukin-6
IU: international unit
K _2_ EDTA: dipotassium ethylenediaminetetraacetic acid
kg: kilogram
LC-MS: liquid chromatography-mass spectrometry
M: mass
mg: milligram
mL: milliliter
mm: millimeter
ng: nanogram
QC: quality control
RPM: revolutions per minute
SCPOR: Saskatchewan Centre for Patient-Oriented Research
SDG: secoisolariciresinol diglucoside
TNF-α: tumor necrosis factor-alpha
µL: microliter
μm: micrometer
